# Mindful creativity: the influence of mindfulness meditation on creative thinking

**DOI:** 10.3389/fpsyg.2013.01020

**Published:** 2014-01-10

**Authors:** Viviana Capurso, Franco Fabbro, Cristiano Crescentini

**Affiliations:** ^1^Department of Psychology, University of Rome “La Sapienza”Rome, Italy; ^2^Department of Human Sciences, University of UdineUdine, Italy

**Keywords:** mindfulness meditation, creativity, insight problem solving, open monitoring, focused attention

Recently the interest in the neurocognitive and psychological effects of meditation, and in particular mindfulness-meditation (MM), has largely grown. From the eighties, research has focused on health benefits of MM and on the cognitive and emotional changes resulting from practice (Kabat-Zinn, 1994; Cahn and Polich, 2006). Several findings suggest that the “observe and accept” approach of MM may lead, among others, to better executive functioning and attention regulation abilities (Jha et al., 2007; Heeren et al., 2009; Moore and Malinowski, 2009; Zeidan et al., 2010).

A new research line investigates the effects of MM on creativity (Ren et al., 2011; Greenberg et al., 2012; Ostafin and Kassman, 2012). In the first of these studies, creative thinking was compared with logic thinking, examining insight and non-insight problem solving, in individuals with different levels of dispositional mindfulness or undergoing brief MM trainings and a specific positive influence of MM on insight problem-solving was found. This effect was interpreted in the light of MM helping to reduce the influence of habitual verbal-conceptual processes on the analysis of ongoing experience, an account in line with more general roles for MM in terms of “erosion” of habitual patterns of responding (Chambers et al., 2008). Insight problem-solving is in fact a class of problems where non-habitual responses or intuition are key factors leading to problem solution (Schooler et al., 1993; Shirley and Langan-Fox, 1996).

Colzato et al. (2012) provided an important contribution to the issue of meditation and creativity. These authors investigated in expert healthy meditators the effects of Focused Attention meditation (FA) and Open Monitoring meditation (OM) on divergent and convergent creative thinking. During FA meditation, participants had to focus attention to particular parts of the body while during OM meditation, they had to open the mind to any occurring thought or sensation, accepting the latter with a non-judgmental attitude. Of importance, FA and OM meditation are both implicated in commonly used MM trainings such as the mindfulness-based stress reduction (Kabat-Zinn et al., 1985; Lutz et al., 2008).

Colzato et al. used the Alternate Uses Task (AUT) for divergent thinking and the Remote Associates Task (RAT) for convergent thinking. In the AUT the participants had to list “as many possible uses for six common household items” while in the RAT they had to find a common associate within three unrelated words. Different versions of the two tasks were administered to each participant after each one of three 35-min FA, OM, or baseline sessions (separated by 10 days). In the latter, the subjects had to visualize a series of household activities. Perceived mood was also assessed with a visual analog scale, showing enhancement of positive mood after OM and FA meditation as compared to baseline.

In line with predictions, Colzato et al. found that engaging in OM meditation, that is likely to bias toward a cognitive-control state characterized by weak and “distributed” top-down control over upcoming thoughts, facilitated performance specifically in the AUT that also requires weak top-down guidance during generation of new ideas which may represent appropriate task solutions. Indeed, after OM meditation participants showed more flexibility, fluency, and originality in their responses. Contrary to predictions, however, to be biased through FA meditation toward strong, focused top-down control over selected thoughts, did not facilitate performance in the RAT that would also require a focused control style operating to constrain the search space of the solution.

Colzato et al. suggested that OM meditation promotes a “distributed” cognitive-control state that should reflect in a broader allocation of cognitive resources leading to better distributed-attention. Nonetheless, in line with previously reported findings of negative influence of mood on convergent thinking (Akbari Chermahini and Hommel, 2012), they hypothesized that improved mood observed after FA meditation could have hampered RAT performance. We believe this to be an intriguing possibility that should be further investigated by also taking into consideration the results of those previous studies that more generally examined the role of attention in creativity. For example, while sustained attention or attention-switching have recently been shown to positively influence insight problem-solving (Murray and Byrne, 2005; Ren et al., 2011), other studies suggested that attention itself may be an obstacle to creative thinking, being this associated to states such as dreaming and reverie (Mendelsohn, 1976; Martindale, 1999; Wierda et al., 2010).

A few other issues deserve discussion. Only experienced practitioners were included in Colzato et al. Hence, it would be interesting to see if the same effects can be observed in naïve meditators for whom the biasing of a distributed cognitive-control state by OM meditation could be less evident. Similarly, relative to expert meditators, naïve individuals could be more hindered by habitual verbal-conceptual processes during AUT-like task solution. Moreover, given other studies on MM and creativity have compared creative thinking with logic thinking (Ren et al., 2011; Ostafin and Kassman, 2012), it would be important to test whether training in FA (or OM) meditation influences insight problem-solving as well as logic thinking. On this view, future studies on MM and creativity considering a larger variety of creativity measures could also try to link creativity performance with different attention tasks (distributed, focused, shifting) (e.g., Jha et al., 2007). Capitalizing on Colzato et al., one could try to verify longitudinally the effects of an 8-week MM training on creativity. Although FA and OM abilities would be interchangeably trained by such an intervention (Lutz et al., 2008), this would allow to investigate longer lasting trait-mindfulness (as compared to state-mindfulness) changes (Cahn and Polich, 2006). Finally it would be interesting to investigate if the effect on creativity is restricted only to performance directly after meditating. This should foster our knowledge on the possible cognitive mechanisms, such as improved mood, better (focused or distributed) attention, or more efficient overcoming of the interference of past experience, through which MM influences creative thinking.

To conclude, Colzato et al. opened a new and interesting scenario that requires further studies to focus on causes of implemented creativity in experienced and naïve meditators, maybe matching long and brief MM trainings to see differences and effects.
